# The Big-Fish-Little-Pond Effect on Academic Self-Concept: A Meta-Analysis

**DOI:** 10.3389/fpsyg.2018.01569

**Published:** 2018-08-29

**Authors:** Junyan Fang, Xitong Huang, Minqiang Zhang, Feifei Huang, Zhe Li, Qiting Yuan

**Affiliations:** Scool of Psychology, South China Normal University, Guangzhou, China

**Keywords:** big-fish-little-pond effect, student, academic self-concept, age, meta-analysis

## Abstract

The Big-fish-little-Pond effect is well acknowledged as the negative effect of class/school average achievement on student academic self-concept, which profoundly impacts student academic performance and mental development. Although a few studies have been done with regard to this effect, inconsistence exists in the effect size with little success in finding moderators. Here, we present a meta-analysis to synthesize related literatures to reach a summary conclusion on the BFLPE. Furthermore, student age, comparison target, academic self-concept domain, student location, sample size, and publication year were examined as potential moderators. Thirty-three studies with fifty-six effect sizes (total *N* = 1,276,838) were finally included. The random effects model led to a mean of the BFLPE at β = −0.28 (*p* < 0.001). Moreover, moderator analyses revealed that the Big-Fish-Little-Pond effect is an age-based process and an intercultural phenomenon, which is stronger among high school students, in Asia and when verbal self-concept is considered. This meta-analysis is the first quantitative systematic overview of BFLPE, whose results are valuable to the understanding of BFLPE and reveal the necessity for educators from all countries to learn about operative means to help students avoid the potential negative effect. Future research expectations are offered subsequently.

## Introduction

In educational psychology, Academic Self-Concept (ASC) refers to students' self-perception in specific disciplines (e.g., math self-concept, science self-concept) or more general academic areas (i.e., global/general ASC) (Marsh et al., 2008a). As a prominent construct in educational psychology, student ASC showed substantial positive relations with many desirable educational outcomes, such as academic effort (Traütwein et al., 2006), academic interest and long-term educational attainment (Marsh et al., 2005, 2007; Pinxten et al., 2010). Earlier empirical researches and a meta-analysis manifested that academic achievement and ASC are reciprocally related (Guay et al., 2003; Valentine and Dubois, 2005; Marsh and Craven, 2006). Positive ASC is an important means of facilitating student academic accomplishments and has been regarded as one of the key objectives of education (Seaton et al., 2009), therefore delving into the ASC forming process and revealing the forming mechanism make an impact both academically and practically.

The Big Fish Little Pond Effect (BFLPE) is one of the most influential theories about student ASC forming process, which was proposed by Marsh (1984) to describe the phenomenon that students in selective schools always have lower ASC compared to those with comparable ability but attend regular schools, which means that being a big fish in a small pond does good to one's ASC. Considerable evidence substantiated that the BFLPE is thought to be the outcome of individuals comparing their ability with the average ability of their group (Marsh, 1987; Plieninger and Dickhäuser, 2015).

It has been demonstrated that student's ASC is shaped not only by his or her performance but also by social comparisons (Marsh, 1988; Marsh et al., 1995; Möller et al., 2009; Parker et al., 2013; Niepel et al., 2014). Students compare their own achievement with that of their class- or schoolmates, which leads them to feel more negative about their own competencies in high-achieving atmosphere than in low-achieving atmosphere. Marsh (1987) argued that this social comparison mechanism lies at the heart of the BFLPE.

Evidence accumulated for several decades supported the BFLPE (Marsh and Hau, 2003; Huguet et al., 2009; Chiu, 2012; Becker and Neumann, 2016; Areepattamannil et al., 2017). The BFLPE was proved to be intercultural and stable: Marsh and Hau (2003) found that the effect of school-average achievement on student ASC is negative in 26 countries ($\bar{\beta }$ = −0.20, *SD* = 0.08), and it exhibits across all student ability levels. Besides, the BFLPE was also observed for students who were at the end of high school or even graduated 2 years or 4 years later (Marsh et al., 2007), students with special education needs (Marsh and Craven, 2006), and students who were identified as gifted (Preckel et al., 2008).

While the BFLPE generally occurs, there are exceptions. Researches by Sung et al. (2014) and Liou (2014) provided evidence for no BFLPE. And results on the size of the BFLPE have been largely mixed. The size of this negative effect ranges from extremely weak (Thijs et al., 2010; Liou, 2014; Becker and Neumann, 2016), to weak (Nagengast and Marsh, 2012; Marsh, 2016) and to moderate (Huguet et al., 2009; Chiu, 2012). These inconsistencies in the reported findings make it difficult to draw a general conclusion concerning the BFLPE and provide useful suggestions for educational practice. As it usually makes more sense to summary existing researches than doing further research (Card, 2012), it is of great importance to carry out a systematic review of the BFLPE. While Marsh et al. (2008b) have summarized the theoretical model underlying the BFLPE, there still lacks quantitative summary in this field.

Discrepancies in reported results provide sufficient incentive for a meta-analysis, and also suggest that there might exist moderating factors accounting for different links. Identifying constructs that may moderate the BFLPE can help further BFLPE theory (Seaton et al., 2009), while little progress has been made in finding factors that strengthen or weaken this effect. Hence, the principal focus of the present investigation is to examine potential moderating variables.

Related results indicated that there may exist one or more variables moderating the BFLPE, such as student age, comparison target, and ASC domain. The first is student age. Marsh (1987) proposed that the BFLPE is more likely to occur when young children begin to form ASC, and Becker and Neumann (2016) supposed that older students are capable enough to deal with conflicting information obtained from contexts, so that they may not suffer the BFLPE. Subjects from a wide range of age groups have been included in BFLPE researches completed to date. Some researchers focused on 15-year-olds from the Programme for International Student Assessment (PISA) (e.g., Nagengast and Marsh, 2012; Marsh, 2016), some took sample of students at grade 4 and grade 8 from the Trends in International Mathematics and Science Study (TIMSS) (e.g., Chiu, 2012; Liou, 2014), and others assessed independent samples at different ages. They usually came out with different results. In Marsh's 2016 study, 276,165 students from PISA 2003 led to the BFLPE at −0.30, while in Preckel's study carried out in 2010, which took a sample of 722 primary school students got a weaker effect (−0.19). Liou (2014) found that the BFLPE was stronger in 8th grade students than 4th grade students, but he didn't do further moderating analysis. The second is the comparison target. In BFLPE researches, students' comparison target was assumed to be a generalized other (Marsh et al., 2008b), which was operationalized by either class-average achievement (e.g., Huguet et al., 2009; Marsh et al., 2009; Preckel and Brull, 2010; Thijs et al., 2010) or school-average achievement (e.g., Seaton et al., 2009; Chiu, 2012; Marsh, 2016; Areepattamannil et al., 2017), and the results varied accordingly. Areepattamannil et al. (2017) assessed the school effect and got the BFLPE at −0.43, while Preckel and Brull (2010) took the class-average achievement as comparison target and got a weaker effect (−0.19). The third is ASC domain. Among the numerous researches about ASC in the BFLPE, some focused on general ASC (e.g., Marsh et al., 2008b; Albert and Dahling, 2016), while others were interested in domain-specific ASC (e.g., Huguet et al., 2009; Jansen et al., 2014), and the size of the effect varies correspondingly. For example, Marsh et al. (2008b) measured general ASC and math ASC in two independent samples simultaneously, while the former got the effect of −0.20, and the latter was −0.44.

In addition to above-mentioned three potential moderators, other study characteristic variables, such as sample size, publication year and student location that have been examined in many published meta-analysis articles were also included in the moderation analyses. Summing up, six potential moderators would be examined in this meta-analysis: student age, comparison target, ASC domain, sample size, publication year, and student location.

We present the first Meta-analysis of the BFLPE synthesizing previous researches on the BFLPE to: (1) provide an integrated effect size of the BFLPE; (2) investigate whether the size of BFLPE will change accordingly when student age changes; (3) find out whether taking class-average achievement as comparison target will lead to different effect size compared with taking school-average achievement as reference; (4) explore the influence of ASC domain on the size of BFLPE; (5) other potential moderating variables, such as sample size, publication year and student location were also examined.

## Methods

### Literature search

#### Search strategies

We systematically searched the quantitative studies evaluating the effect of class- or school-average achievement on student ASC. To find all articles that met our criteria, we conducted a literature search using the Educational Database, Research Library, Psychology Database, PsycARTICLES, PsycINFO, and ERIC. Each database was searched using the following key terms: *Big fish little pond* or *academic self-concept* in the abstract and *average* in the full text. We searched for all full-text and peer-review articles written in English and published from January 1st 1984 to January 1st 2018. Because the BFLPE was first put forward by Marsh and Parker (1984). The initial search revealed 386 articles in total.

#### Inclusion and exclusion criteria

Articles were included based on the following criteria: (1) quantitative researches whose topic was the BFLPE on student ASC; (2) used the classic BFLPE model that test the class/school effect after controlling for student effect; (3) explicitly reported the regression coefficients of class/school average achievement on student ASC; (4) provided detailed information about class/school that was taken as the comparison target; (5) results derived from subjects with intellectual disability or learning disability were not considered here.

**Figure 1:**
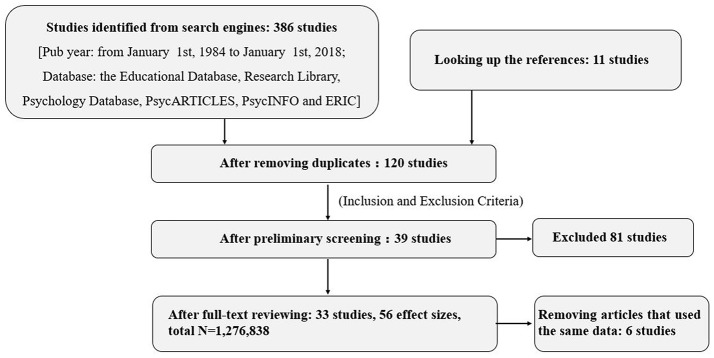
Flow diagram showing the process through the literature search, study selection, and study inclusion for the meta-analysis.

This preliminary selection procedure resulted in 39 studies. After excluding the studies using the same data resource, we got 33 studies in total with 56 effect sizes (*N* = 1,276,838) in the end. The whole process was based on PRISMA and detailed information about the process through literature search, study selection, and study inclusion for the meta-analysis was illustrated in Figure 1.

### Coding procedures

#### Outcome variable

We focus on the effect of class- or school-average achievement on student ASC, so the multilevel regression coefficients β and sample size of each study were recorded.

Regression coefficients were coded based on an independent sample, and separately coded if a study had several independent samples. Besides, if a study included repeated measurement experiments at different time, the result retrieved from the last measurement would be chosen.

#### Potential moderating variables

Six potential moderators would be examined in this meta-analysis: student age, comparison target, ASC domain, sample size, publication year and student location.

These 33 studies were carefully coded for the following variables.

1. Student Age. Student age was coded as “primary school,” “middle school,” “high school,” or “college.”
2. Comparison target. The comparison target was recorded as “school” or “class.”
3. ASC domain. The domain that student ASC was measured was recorded as “general,” “verbal,” or “STEM” (Science, Technology, Engineering, Mathematics). For example, studies using measuring scales that contain statements like “I am good at English/French/Verbal” would be codes as “verbal.”
4. Sample size.
5. Pub-year. The publication year was recorded.
6. Student location. The student location refers to the area where participants come from, it was coded as “Asia,” “Europe,” “North America,” “Oceania,” or “Mix.”

We didn't consider student gender because the BFLPE was tested to be robust over gender (Marsh and Hau, 2003). And the type of measuring tool was not considered because this variable can't be categorized that many researchers just reported the achievement measure as quote from some International Education Survey Project or offered vague information about item type, so we didn't examine its moderating effect here. The coding was conducted by two researchers twice with an interval of 2 months.

### Statistical analysis

#### Effect size

Comprehensive Meta-Analysis software program version 3.0 was used to conduct the meta-analysis. Each regression coefficient was transformed into a Fisher's *Z* score as an effect size (ES), and all weighted mean ESs and corresponding confidence intervals were converted back at last for a better understanding.

#### Heterogeneity

Cochran's *Q*-Test and the *I*^2^ statistic were used for the homogeneity test. Moderator analyses were conducted after the homogeneity test. *I*^2^values of 0–25% were interpreted as no heterogeneity, 25–50% as low heterogeneity, 50–75% as moderate heterogeneity, and 75–100% as high heterogeneity among studies.

#### Publication bias

The funnel plot and Egger regression test were used to test whether the results were biased due to different publication sources.

## Results

### Characteristic of the studies included

Study name (presented as “first author's last name & publication year”), regression coefficient, *N* (sample size), ES (effect size) and student age of each study included are reported in Table 1. Comparison target, ASC domain, and student location are reported in Table 2.

**Table 1:** Summary of studies included in the meta-analysis (1).

**Number**	**Study name**	**Regression coefficient**	***N***	**ES**	**Student age**
1	Arens and Watermann, 2015	−0.28	4,925	−0.29	Primary school
2	Areepattamannil et al., 2017	−0.43	7,404	−0.46	High school
3	Chiu, 2012	−0.50	139,174	−0.55	Middle school
4	Chiu, 2012	−0.28	139,174	−0.29	Middle school
5	Dumont et al., 2017	−0.11	2,155	−0.11	Middle school
6	Dumont et al., 2017	−0.16	2,155	−0.16	Middle school
7	Dumont et al., 2017	−0.12	2,155	−0.12	Middle school
8	Huguet et al., 2009	−0.47	2,015	−0.51	Middle school
9	Huguet et al., 2009	−0.45	2,015	−0.48	Middle school
10	Jansen et al., 2015	−0.28	4,891	−0.29	High school
11	Jansen et al., 2015	−0.10	9,167	−0.10	Middle school
12	Jansen et al., 2015	−0.09	9,167	−0.09	Middle school
13	Liem and Yeung, 2013	−0.31	4,461	−0.32	Middle school
14	Liem and Yeung, 2013	−0.61	4,461	−0.71	Middle school
15	Liem and Yeung, 2013	−0.30	4,461	−0.31	Middle school
16	Liem and Yeung, 2013	−0.29	4,461	−0.30	Middle school
17	Liou, 2014	−0.29	4,284	−0.30	Primary school
18	Liou, 2014	−0.06	4,284	−0.06	Primary school
19	Liou, 2014	−0.29	5,042	−0.30	Middle school
20	Liou, 2014	−0.14	5,042	−0.14	Middle school
21	Lohbeck and Moller, 2017	−0.13	291	−0.13	Primary school
22	Marsh, 1984	−0.27	305	−0.28	Primary school
23	Marsh, 1987	−0.23	2,213	−0.23	High school
24	Marsh, 1990	−0.22	14,825	−0.22	High school
25	Marsh, 1990	−0.22	14,825	−0.22	High school
26	Marsh, 1994	−0.14	4,184	−0.14	High school
27	Marsh, 1994	−0.10	4,184	−0.10	High school
28	Marsh and Rowe, 1996	−0.14	1,628	−0.14	High school
29	Marsh et al., 2000	−0.21	7,997	−0.21	Middle school
30	Marsh et al., 2001	−0.19	2,778	−0.19	Middle school
31	Marsh et al., 2007	−0.28	1,758	−0.29	High school
32	Marsh et al., 2007	−0.21	1,758	−0.21	College
33	Marsh et al., 2008b	−0.20	103,558	−0.20	High school
34	Marsh et al., 2008b	−0.44	736	−0.47	Middle school
35	Marsh and O'Mara, 2010	−0.34	2,213	−0.35	High school
36	Marsh and O'Mara, 2010	−0.14	1,886	−0.14	High school
37	Marsh and O'Mara, 2010	−0.25	1,620	−0.26	College
38	Marsh, 2016	−0.30	276,165	−0.31	High school
39	Nagengast and Marsh, 2011	−0.21	398,411	−0.21	High school
40	Preckel and Brull, 2010	−0.19	722	−0.19	Middle school
41	Parker et al., 2013	−0.60	5,016	−0.69	High school
42	Parker et al., 2013	−0.28	5,016	−0.29	High school
43	Parker et al., 2013	−0.41	5,016	−0.44	High school
44	Parker et al., 2013	−0.67	5,016	−0.81	High school
45	Roy et al., 2015	−0.14	422	−0.14	Primary school
46	Sung et al., 2014	−0.27	5,640	−0.28	High school
47	Scherer and Siddiq, 2015	−0.27	4,686	−0.28	High school
48	Szumski and Karwowski, 2015	−0.40	4,252	−0.42	Primary school
49	Szumski and Karwowski, 2015	−0.23	5,276	−0.23	Primary school
50	Stäbler et al., 2017	−0.10	6,463	−0.10	Middle school
51	Traütwein et al., 2006	−0.76	14,341	−1.00	High school
52	Trautwein et al., 2009	−0.22	4,810	−0.22	High school
53	Trautwein et al., 2009	−0.46	1,502	−0.50	High school
54	Trautwein et al., 2009	−0.23	4,247	−0.23	High school
55	Thijs et al., 2010	−0.09	1,649	−0.09	Primary school
56	Wouters et al., 2011	−0.07	536	−0.07	High school

*We use first author and publication year to represent each study, and if one study provided more than one effect size, we use ①,②,③,④ to indicate*.

*Regression coefficient refers to original coefficient in each study*.

*The formula to get the effect size: $Z_{\beta }=\text{0.5}\times ln\left(\frac{\text{1}+\beta }{\text{1}-\beta }\right)$*.

**Table 2:** Summary of studies included in the meta-analysis (2).

**Number**	**Study name**	**Comparison target**	**ASC domain**	**Student location**
1	Arens and Watermann, 2015	Class	General	Europe
2	Areepattamannil et al., 2017	School	STEM	Asia
3	Chiu, 2012	School	STEM	Mix
4	Chiu, 2012	School	STEM	Mix
5	Dumont et al., 2017	School	STEM	Europe
6	Dumont et al., 2017	School	Verbal	Europe
7	Dumont et al., 2017	School	General	Europe
8	Huguet et al., 2009	Class	STEM	Europe
9	Huguet et al., 2009	Class	Verbal	Europe
10	Jansen et al., 2015	School	STEM	Europe
11	Jansen et al., 2015	Class	Verbal	Europe
12	Jansen et al., 2015	School	Verbal	Europe
13	Liem and Yeung, 2013	Class	STEM	Asia
14	Liem and Yeung, 2013	Class	Verbal	Asia
15	Liem and Yeung, 2013	School	STEM	Asia
16	Liem and Yeung, 2013	School	Verbal	Asia
17	Liou, 2014	School	STEM	Mix
18	Liou, 2014	School	STEM	Mix
19	Liou, 2014	School	STEM	Mix
20	Liou, 2014	School	STEM	Mix
21	Lohbeck and Moller, 2017	Class	STEM	Europe
22	Marsh, 1984	School	General	Oceania
23	Marsh, 1987	School	General	North America
24	Marsh, 1990	School	STEM	North America
25	Marsh, 1990	School	Verbal	North America
26	Marsh, 1994	School	STEM	North America
27	Marsh, 1994	School	Verbal	North America
28	Marsh and Rowe, 1996	School	General	North America
29	Marsh et al., 2000	School	General	ASIA
30	Marsh et al., 2001	Class	STEM	Europe
31	Marsh et al., 2007	School	STEM	Europe
32	Marsh et al., 2007	School	STEM	Europe
33	Marsh et al., 2008b	School	general	Mix
34	Marsh et al., 2008b	Class	STEM	Europe
35	Marsh and O'Mara, 2010	School	General	North America
36	Marsh and O'Mara, 2010	School	General	North America
37	Marsh and O'Mara, 2010	School	Verbal	North America
38	Marsh, 2016	School	STEM	Mix
39	Nagengast and Marsh, 2012	School	STEM	Mix
40	Preckel and Brull, 2010	Class	STEM	Europe
41	Parker et al., 2013	School	STEM	Europe
42	Parker et al., 2013	School	STEM	Europe
43		School	Verbal	Europe
44	Parker et al., 2013	School	Verbal	Europe
45	Roy et al., 2015	Class	Verbal	North America
46	Sung et al., 2014	School	general	Asia
47	Scherer and Siddiq, 2015	School	STEM	Europe
48	Szumski and Karwowski, 2015	Class	General	Europe
49	Szumski and Karwowski, 2015	Class	General	Europe
50	Stäbler et al., 2017	Class	STEM	Europe
51	Traütwein et al., 2006	School	STEM	Europe
52	Trautwein et al., 2009	School	STEM	Europe
53	Trautwein et al., 2009	Class	STEM	Europe
54	Trautwein et al., 2009	School	STEM	Europe
55	Thijs et al., 2010	Class	General	Europe
56	Wouters et al., 2011	Class	General	Europe

*We use first author and publication year to represent each study, and if one study provided more than one effect size, we use ①,②,③,④ to indicate*.

*STEM, refers to Science, Technology, Engineering, Mathematics*.

A total of *N* = 1,276,838 were involved in the included 33 studies, and 56 ESs were coded out of the studies.

Thirty-nine of the ESs were based on Large-scale assessments (7 for PISA, 6 for TIMSS, and 26 for other assessments like TOSCA), other 17 were retrieved from studies collecting data independently.

Seven of the ESs were based on students from Asia (4 for Singapore, 1 for United Arab Emirates, and 2 for Taiwan, China), 29 were based on Europe students (19 for Germany, 3 for Belgium, 2 for France, 1 for Netherlands, 1 for Norway, 2 for Poland, and 1 for UK), 10 were based on North America students, 1 was based on Oceanian students and 9 were Mix (e.g., from 27 countries).

Fourteen of the ESs were based on general ASC, 30 were based on STEM ASC (22 for mathematics ASC, 8 for science ASC), and 12 were based on verbal ASC (4 for French ASC, 6 for English ASC, 2 for general verbal ASC).

### Publication bias

**Figure 2:**
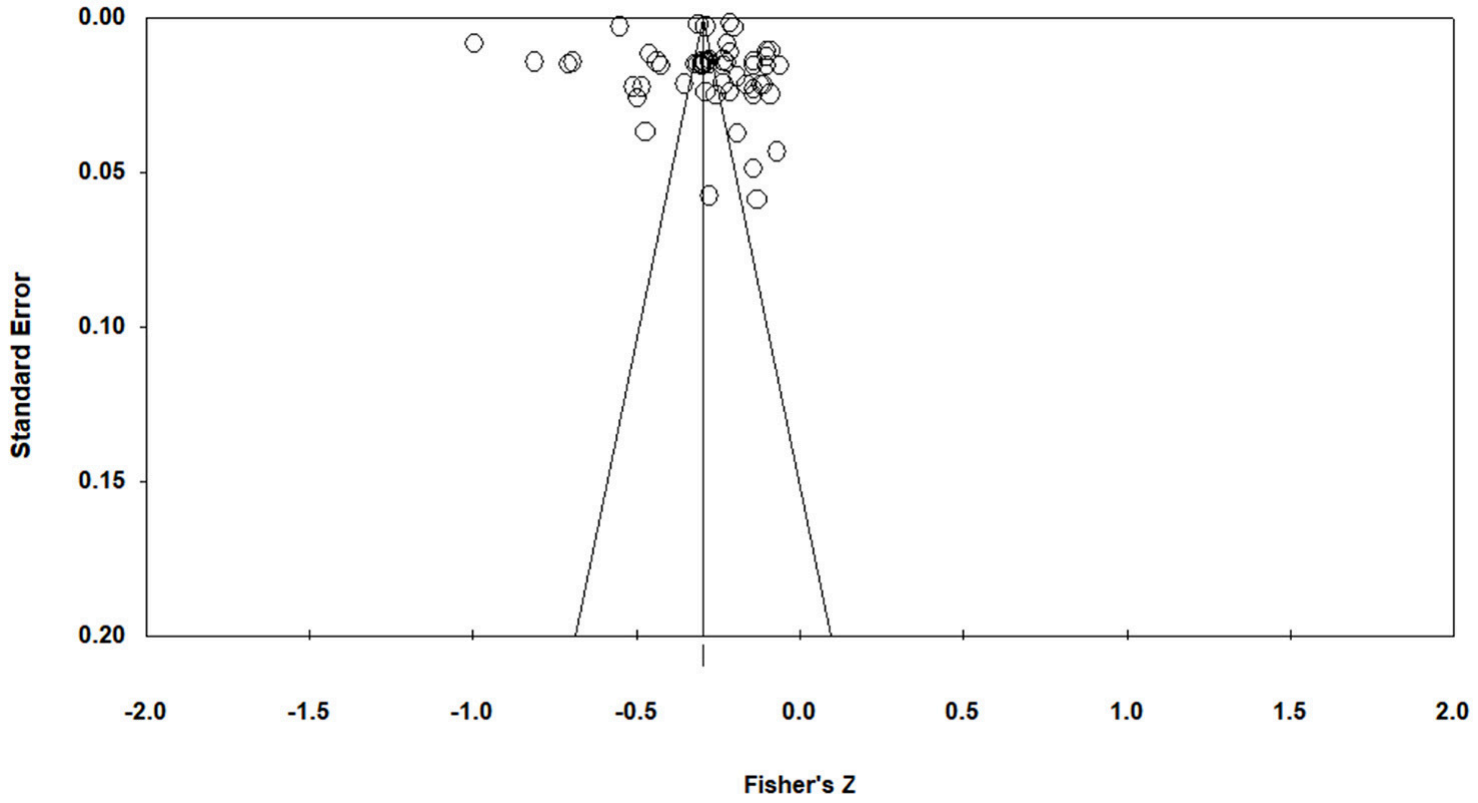
Funnel plot.

As we can see from Figure 2, the Funnel plot showed that all the 56 ESs are evenly distributed on both sides and gather at the top of the plot, and the Egger regression revealed no significant bias with *t* = 0.32 (*df* = 54, *p* > 0.05). Together, we can conclude that the results were not biased due to the publication sources.

### Mean effect size

The homogeneity test results were *Q* = 25,478.88 (*df* = 55, *p* < 0.001), *I*^2^ = 99.78%, so the random effects model was chosen. The integrated results showed a significant negative effect of class/school average achievement on student ASC: β = −0.28 (*Z* = −13.84, *p* < 0.001, 95% CI = [−0.32, −0.24]), which means that students in class/school with an average ability level one standard deviation above the mean have ASC that is 0.28 of a standard deviation below the average ASC level. These effect sizes were suitable for subsequent moderator analyses.

### Moderator analyses

#### Student age

The mixed effects model was chosen here. As showed in Table 3, the main effect of student age was significant: *Z* = −17.56, *p* < 0.001, and the heterogeneity test was significant with *Q* = 7.86 (*df* = 3, *p* < 0.05), which meant that student age significantly moderates the BFLPE. From Table 3, we can also see that students in high school indicate the strongest effect (β_highschool_ = −0.32), while middle school and college students show a moderate effect (β_middleschool_ = −0.28, β_college_ = −0.23), and primary school students show the weakest effect (β_primaryschool_ = −0.21). These results indicated that the BFLPE is the strongest when students in high school, weaker in middle school and college, and shows the weakest in primary school.

**Table 3:** Student age as moderator of the BFLPE.

**Moderator**	***k***	**β**	**95% CI**		***Z***
			**LL**	**UL**	
Student age					−17.56^***^
Primary school	9	−0.21	−0.29	−0.29	−4.98^***^
Middle school	20	−0.28	−0.35	−0.26	−6.61^***^
High school	25	−0.32	−0.37	−0.20	−11.19^***^
College	2	−0.23	−0.27	−0.13	−11.07^***^

CI, Confidence Interval; ^*^p < 0.05, ^**^p < 0.01,

*** *p < 0.001*.

#### Comparison target

There was no significant influence of comparison target: *Q* = 0.01 (*df* = 1, *p* > 0.05), which meant that whether the study takes class-average achievement or school-average achievement as comparison target has little influence on the size of BFLPE.

#### Academic self-concept domain

As showed in Table 4, the main effect of ASC domain was significant: *Z* = −15.62, *p* < 0.001, and the heterogeneity test was significant with *Q* = 7.23 (*df* = 2, *p* < 0.05), which meant that ASC domain significantly moderates the BFLPE. From Table 4, we can also see that verbal ASC indicates the strongest effect (β_verbalASC_ = −0.31), while STEM ASC shows moderate effect (β_STEMASC_ = −0.30), and general ASC shows the weakest effect (β_generalASC_ = −0.22). These results indicated that the BFLPE varies with the domain of ASC and indicates strongest when verbal ASC is considered.

**Table 4:** ASC domain as moderator of the BFLPE.

**Moderator**	***k***	**β**	**95% CI**		***Z***
			**LL**	**UL**	
ASC domain					−15.62^***^
General ASC	14	−0.22	−0.26	−0.18	−10.89^***^
STEM ASC	30	−0.30	−0.35	−0.25	−10.61^***^
Verbal ASC	12	−0.31	−0.43	−0.18	−4.48^***^

CI, Confidence Interval; ^*^p < 0.05, ^**^ p < 0.01,

*** *p < 0.001*.

#### Sample size

Meta-regression showed that there was no significant influence of sample size with *Q* = 0.00 (*df* = 1, *p* > 0.05).

#### Publication year

Meta-regression showed that there was no significant influence of publication year with *Q* = 0.35 (*df* = 1, *p* > 0.05).

#### Student location

As showed in Table 5, the main effect of student location was significant: *Z* = −14.56, *p* < 0.001, and the heterogeneity test was significant with *Q* = 11.07 (*df* = 4, *p* < 0.05), which meant that student location significantly moderates the BFLPE. From Table 5, we can also see that Asian students indicate the strongest effect (β_Asia_ = −0.35), while North American students show the weakest effect (β_NorthAmerica_ = −0.20), and students in Europe, Oceania and mixed countries show the moderate effect (β_Europe_ = −0.30, β _Oceania_ = −0.27, β_Mix_ = −0.26). These results indicated that the BFLPE varies with student location of participants and indicates strongest in Asia.

**Table 5:** Student location as moderator of the BFLPE.

**Moderator**	***k***	**β**	**95% CI**		***Z***
			**LL**	**UL**	
Student location					−14.56^***^
Asia	7	−0.35	−0.45	−0.25	−6.04^***^
Europe	29	−0.30	−0.40	−0.20	−5.44^***^
North America	10	−0.20	−0.23	−0.16	−9.75^***^
Oceania	1	−0.27	−0.37	−0.16	−4.81^***^
Mix	9	−0.26	−0.33	−0.18	−6.22^***^

CI, Confidence Interval; ^*^p < 0.05, ^**^p < 0.01,

*** *p < 0.001*.

## Discussion

### The BFLPE

As the first meta-analysis of the BFLPE, this paper presents a new perspective into this theory and provides a reliable synthesized result of the effect size of the BFLPE based on empirical researches. More importantly, six potential moderators were examined and student age was found to significantly moderate the BFLPE.

The combined results show a significant negative effect of class/school average achievement on student ASC: β = −0.28 (*Z* = −13.84, *p* < 0.001, 95% CI = [−0.32, −0.24]), which means that students in class/school with an average ability level one standard deviation above the mean have ASC that is 0.28 of a standard deviation below the average ASC level. The result confirms that the BFLPE is prevailing and robust in educational psychology, as supported by many other cross-culturable empirical studies (Marsh et al., 2014, 2015; Marsh, 2016).

The results of the meta-analysis contribute to the BFLPE realm both theoretically and practically. First of all, confirmation of the persistence of the BFLPE demonstrates the point that students' perception of oneself can be understood in consideration of social comparison theory, which argues that unpleasant social comparison experienced in higher ability educating environment may induce lower ASC (Marsh et al., 1995; Huguet et al., 2009). Since there lacks less able students to make favorable comparison with and overflows with more able students in a highly capable group, it is possible for students to experience uncertainty about one's own ability and ambiguity in verifying their own competence, which may induce lower ASC. Second, the BFLPE could give explanations for educational phenomena. For example, average students in general classes or schools always have more positive ASC than those abler ones attending advanced placement, which can be interpreted by the BFLPE that the former usually rank favorably in their local environment, while the latter frequently rank unfavorably with much more high-quality peers in their surroundings. Last but not least, negative consequences of being in a more competitive educational setting should not be ignored. From the perspective of parents who consider sending their children to high-achieving schools or transferring children to advanced classes, they should be informed of the potential negative consequence on ASC; as for educators, understanding how ASC might be influenced by the BFLPE can facilitate application of appropriate teaching practices, so that they can help students develop proper ASC, which is necessary for fine academic development. It has been demonstrated that differentiated instruction strategies can be used to attenuate the BFLPE (Roy et al., 2015); besides, it reveals the necessity of special education classes or schools: when disadvantaged students are put in regular schools/classes, they are very likely to suffer from low ASC for being small fishes in the big pond.

### Moderating role of student age

The BFLPE was found significant in all age groups in this study, from primary school to college, which coincides with the point that the BFLPE is more likely to occur in elementary (primary) school, during when children are in the formatting self-concepts (Marsh, 1987).

Moreover, this meta-analysis found that student age significantly moderates the BFLPE, that is, the BFLPE is the strongest when students in high school, weaker in middle school and college, and shows the weakest in primary school. It coincides with past assumptions that inferring a person's ability is a process underlying ASC, and only those who have developed the most differentiated conceptions of ability are able to infer other's ability based on their achievement and efforts (Marsh, 1984). Besides, social comparison that plays an important role in the BFLPE largely correlates with cognitive development.

Early adolescents, as primary school students in this study, begin to master social comparison, but still lack the ability to integrate different information about themselves (Harter, 2003), so they show a significant BFLPE but very small in size. As their cognitive skills and academic pressure grow, the effect size increases a bit in middle school. Students in college are old and experienced enough to get rid of relying too much on others, which means that they are capable to assess their own academic skills independent of the performance of their classmates (Marsh, 1987; Becker and Neumann, 2016), so the decline happens in the BFLPE. As for high school students' strongest effect, we can explain it in two ways. First, the tracking effect. Academic tracking system has been the most-implemented curriculum delivery model in almost all schools, which mostly happens during high school (Lüdtke et al., 2006; Falkenstein, 2007; Liu and Wang, 2008; Wouters and Fraine, 2010; Houtte and Stevens, 2015; Salchegger, 2016; Dumont et al., 2017). The academic tracking system divides students into class/school levels for low, medium, and high achievers in each grade based on past performance, which may increase the chances of experiencing unpleasant comparison for students in intermediate-track or high-track schools; second, high school students are experiencing a period of life characterized by increased self-consciousness, and they always face more academic pressure. So synthetically considering, students in this age group would be much more influenced by the class/school-average ability.

These results suggest that the BFLPE is an age-based process, which occurs at primary school age and reaches peak value during high school. Considered that ASC in high school has been found to be more salient than actual academic achievement in predicting learning effort, educational and occupational aspirations, and subsequent university course selection (Guay et al., 2004; Marsh et al., 2008a), special caution from teachers and parents should be paid for high school students, who are at risk of suffering the strongest BFLPE.

### Moderating role of academic self-concept domain

The BFLPE was found significant in all three domains of ASC and the size of the BFLPE was found to vary by different ASC domains: general ASC resulted the lowest effect, verbal ASC showed the strongest effect, and STEM ASC indicated medium effect.

In 1976, Shavelson, Hubner, and Stanton presented the Shavelson model (cf. Byrne and Worth Gavin, 1996), which posited ASC to be hierarchically organized, with general ASC at the apex of the hierarchy. Empirical researches strongly support the hypotheses of the hierarchical organization (Marsh et al., 1988; Marsh, 1990; Martin et al., 2010). General ASC is regarded as relatively stable competence beliefs that is independent of the situation (e.g., Scherbaum et al., 2006). Besides, general ASC is found to directly influences domain-general and subject-specific measures of ASC. Hence, general ASC directly accounts for a substantial amount of variance in all measures of ASC (Martin et al., 2010). Summing up the above, general ASC has the ability to maintain relative stability, so it may suffer less from the negative effect of class/school average achievement.

There exists clear distinction between verbal ASC and STEM ASC (Marsh, 1986). Compared with STEM ASC, verbal ASC exposes more to external comparison. Generally speaking, various language activities will be held in class or school, which will bring rich success-failure experience, so that students more frequently compare their own verbal abilities with the perceived abilities of other students in their frame of reference and use this external impression as one basis of their self-perceptions of verbal ASC. Besides, external observers usually form the evaluation of one's verbal ability based on their speaking skills, which in turns lead to change in verbal ASC. Thus, verbal ASC may be more easily influenced by the average ability of classmates or schoolmates, which will show the strongest BFLPE.

### Moderating role of student location

The BFLPE was found significant in all student locations here, which verifies the BFLPE is intercultural and stable (Marsh and Hau, 2003). The result also reveals that learning to avoid the negative effect of the BFLPE is necessary for educators from all countries.

Besides, the size of BFLPE was found to be strongest for Asian students and weakest in North America. Asian participants here were most from Taiwan, China and Singapore, which are highly industrialized and always perform outstandingly in international large-scale assessments (Liou, 2014). The possible reason for the strongest relation between class/school-average achievement and ASC may be the cultural difference. Seaton et al. (2009) put forward that the size of BFLPE varies across countries and the different population may lead to different patterns between student ASC and achievement (Liou, 2014). Most Asian students are raised up in surroundings highly value academic achievement while students from other student locations face less academic stress than Asian ones, and Asian schools always emphasize the competition with their peers, so they may compare with classmates and schoolmates more frequently, besides, Asian students are found to have a high level of test anxiety and self-doubt compared with their counterparts (Stankov, 2010), which result in the strongest BFLPE in Asian students.

The non-significant moderating effect of sample size, and publication year reveal that the size of the BFLPE doesn't vary as sample size or publication year changes, which confirm the BFLPE's universality and robustness (Marsh et al., 2014, 2015; Marsh, 2016).

### Limitations

There exists an apparent gap between the number of different comparison targets (39 for school-average achievement and 17 for class-average achievement). This may result in the insignificant result in the moderation analyses, so future research can broaden the scope of literature search to obtain enough studies. Furthermore, the dependence of ESs caused by deriving more than one ES from a study or from studies conducted by the same research team was not examined here, which can be further discussed using a multilevel model.

### Future research

Regarding the direction of future research, the possible moderating role of student ability can be taken into consideration. Although the BFLPE was found in students across different level of ability (Marsh and Hau, 2003), some researches (Marsh and Rowe, 1996; Trautwein et al., 2009) found that the ASC of relatively high-achieving students appear to be less affected by BFLPE than those of relatively low-achieving students. Roy et al. (2015) also found that significant BFLPE was only for students with low individual achievement and for whom teachers reported less frequent use of differentiated instruction strategies. So, it is worth exploring whether the BFLPE is moderated by students' ability level.

## Conclusion

This research made these main contributions: (1) presents a new perspective of the BFLPE by conducting a meta-analysis, which goes beyond prior work by providing a reliable quantitative conclusion of the BFLPE; (2) examines six potential moderating variables and identifies three moderators of the BFLPE: student age, student location and ASC domain. The findings help further the understanding of the BFLPE and make it clear that BFLPE is an age-based process, which occurs at primary school age and reaches peak value during high school. Besides, the BFLPE varies with student location and ASC domain, indicating strongest when verbal ASC is considered and for Asian students. Furthermore, these findings have utility for educators. A better understanding of these processes may enable teachers to better motivate students and provides credible reinforcement to seek measures to reduce the negative BFLPE.

## Author contributions

JF, XH, and MZ came up with the experiment ideas. JF and FH did literature research. JF, XH, and ZL analyzed experimental results. JF and QY wrote the manuscript.

### Conflict of interest statement

The authors declare that the research was conducted in the absence of any commercial or financial relationships that could be construed as a potential conflict of interest.
